# Intrahepatic cholangiocarcinoma as a unique subtype: key updates from current guidelines

**DOI:** 10.1007/s00432-025-06342-3

**Published:** 2025-10-25

**Authors:** Aaron Schindler, Timm Denecke, Daniel Seehofer, Florian van Bömmel, Thomas Berg

**Affiliations:** 1 Division of Hepatology, Department of Medicine II, Leipzig University Medical Center, Leipzig, Germany; 2 Department of Diagnostic and Interventional Radiology, Leipzig University Medical Center, Leipzig, Germany; 3 Department of Visceral, Thoracic and Vascular Surgery, Leipzig University Medical Center, Leipzig, Germany; 4 University Liver Tumor Center (ULTC), Leipzig, Deutschland; 5 Leipzig University Medical Center, Leipzig, Germany; 6 Laboratory for Clinical and Experimental Hepatology, Division of Hepatology, Department of Medicine II, Leipzig University Medical Center, Leipzig, Germany

**Keywords:** Liver cancer, Intrahepatic Cholangiocarcinoma, Overview current guidelines

## Introduction

Cholangiocarcinoma (CCA) is the second most common primary liver cancer after hepatocellular carcinoma (HCC), accounting for approximately 10–15% of all primary liver cancers (Banales 2020). Biliary tract cancers (BTC) comprise a heterogeneous group of invasive adenocarcinomas originating from the gallbladder or cystic duct (GBC), and the biliary tree (CCA) (ESMO Guideline 2023). CCA is further subclassified into intrahepatic (iCCA), perihilar (pCCA), and distal (dCCA) subtypes, based on their anatomical location (EASL-ILCA Guideline 2023). ICCA arises between the bile ductules and the second-order bile ducts.

**Fig. 1:**
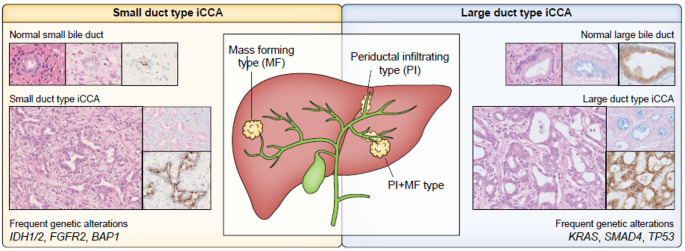
Macroscopically and microscopically classification of intrahepatic cholangiocarcinoma (iCCA). iCCA is divided into two subtypes: „small duct type “ and „large duct type “, that differ regarding to growth and genetic alterations (taken from EASL-ILCA-Guideline 2025). Reprinted with permission by Elsevier (Ref.Nr. 6,101,370,428,404)

Importantly, iCCA differs markedly from other BTC subtypes in terms of etiology, risk factors, molecular alterations, and clinical management (EASL-ILCA Guideline 2023). Molecular profiling of tumor tissue further highlights the biological distinctiveness of iCCA, which reflects not only its anatomical location but also a unique and heterogenous molecular signature (Fig. 1). Early diagnosis, molecular characterization, accurate staging and personalized interdisciplinary management remain key challenges in the treatment of iCCA. Given the heterogeneity of existing guidelines for the treatment of CCA, this short review provides a focused summary of current clinical recommendations (ESMO Guideline 2023; EASL-ILCA Guideline 2023; Deutsche S3-Leitlinie 2024) for the management of iCCA with particular emphasis on the EASL-guideline, the first to offer a dedicated framework specifically for iCCA.

## Epidemiology and classification

The age standardised incidence rate of CCA remains low in Europe, the USA and Australasia ranging from 0.3 to 3.5 cases per 100,000 population (ESMO Guideline 2023). Despite this, the global mortality rate for CCA has increased over recent decades, as reported by the World Health Organization and Pan American Health Organization databases (ESMO Guideline 2023; Bertuccio 2019). While overall CCA incidence in Asia has remained stable or declined, the incidence of iCCA has shown a steady rise in most Western countries (ESMO Guideline 2023; Patel 2002; Saha 2016; Khan 2019). Age is a continuos and cumulative risk factor (Deutsche S3-Leitlinie). The highest CCA incidences are observed in south east Asia where liver fluke infections cause chronic cholangitis with local rates reaching up to 80 persons per 100,000 population (Banales 2016).

## Classification—macroscopically

iCCA is classified into four subtypes: (a) „mass-forming “ (MF), (b) periductal-infiltrating (PI), (c) MF + PI and (d) intraductal growing (EASL-ILCA Guideline). The latter is also categorised by WHO as intraductal papillary neoplasm (EASL-ILCA Guideline). The distinction between mass-forming or periductal-infiltrating is clincially relevant as the periductal-infiltrating subtype is associated with a poorer prognosis and a higher frequency of lymphatic and perineural invasion (EASL-ILCA 2023; Liau et al. 2014; Watanabe et al. 2020; Ohtsuka et al. 2002; Hirohashi et al. 2002; Isaji et al. 1999).

### Classification – microscopically/histologically: „large duct type “ und „small duct type “

iCCA is further subclassified into a „small duct type “ and a „large duct type “. The large duct type typically exhibits glandular structures with mucine production and is associated with diseases of the bile ducts such as primary sclerosing cholangitis (PSC), hepatolithiasis or liver fluke infection (EASL-ILCA Guideline 2023). In contrast, the small duct type is more commonly linked to non-biliary chronic liver diseases including viral hepatitis and metabolic dysfunction-associated steatotic liver disease (MASLD) (EASL-ILCA Guideline 2023). Long term outcomes are generally more favorable in small duct type compared to the large duct type (Liau et al. 2014; Akita et al. 2017; Hayashi et al. 2016). This subclassification also has therapeutic implications: the small duct type frequently harbours isocitrate dehydrogenase (IDH)-1 mutations and fibroblast growth factor receptor (FGFR) fusions, both of which represent actionable molecular targets (Table 1) (EASL-ILCA 2023). Notably, the small duct type presents exclusively with a mass-forming growth pattern, whereas the large duct type is typically periductal-infiltrating or exhibits a combined mass-forming and periductal-infiltrating pattern (EASL-ILCA 2023).

**Table 1:** Typical features of iCCA-subtypes, adapted from Deutsche S3-Leitlinie 2024.^4^

Criteria	Small-duct type	Large-duct type
Predisposing disease	Chronic hepatitis B/C, MASLD, chronic liver disease, cirrhosis	PSC, liver fluke, bile duct lithiasis
Premalign lesion	Unknown	BilIN, IPNB, MCN
Macroscopically	Mass forming	Periductal infiltrating
Molecular changes	p53, KRAS, FGFR-2, IDH 1 + 2	KRAS, p53, ARID1B, SMAD4
Therapeutical targets	IDH1 + 2, FGFR-2, NTRK, BRAF-mut, MSIhigh	BRCA-1/-2, Her-2-Amp, MSIhigh

## Risk factors

### Risk factors for developing iCCA

In up to 60–70% of iCCA diagnosis, the predisposing risk factor is not detectable. This leads to late diagnosis in advanced stages. Risk factors for CCA share chronic inflammation of the biliary epithelium as a key feature (ESMO Guideline 2023; Khan et al. 2019). Diabetes, obesity and use of hormonal contraceptives have been associated with an 81%, 62% and 62% increase of risk of iCCA, respectively (ESMO Guideline 2023; Petrick et al. 2018; Petrick et al. 2020). There are only some identified subgroups, that benefit from surveillance (Table 2).

**Table 2:** Risk factors for iCCA and odds ratio (OR), adapted from EASL-ILCA Guideline and Gudbjartsson et al.

Risk factors for ICC	OR
*Choledochal cyst*	**26.71**
*Choledocholithiasis*	**10.08**
*Caroli disease*	**38**
*Primary sclerosing cholangitis*	**22**
*Chronic hepatitis B*	**4.57**
*Chronic hepatitis C*	**4.28**
*MDR3 variants of hepaticocanalicular transporters*	**3–5**
*Haemochromatosis*	**2.1**
*Metabolic dysfunction-associated steatotic liver disease (MASLD)*	**2.2**
*Liver fluke*	**2**
*Alcohol consumption*	**3.15**

### PSC

In western countries PSC is the most significant risk factor for the development of CCA (odds ratio (OR) 20–25 for iCCA) (EASL-ILCA 2023; Yoon et al. 2021; Ma et al. 2020; Chung et al. 2021; Akita et al. 2019; Ahn et al. 2019). Current EASL-guidelines recommend screening annualy with MRI/MRCP and carbohydrat antigen (CA) 19–9. The surveillance modality with the best specificity and sensitivity is MRI. The german guideline recommends surveillance every 6 month alternating with MRI and ultrasound (Deutsche S3-Leitlinie 2024).

### Cirrhosis

Liver cirrhosis of any aetiology is a significant risk factor for the development of iCCA with reported odds ratios ranging from 9 to 25 (EASL-ILCA Guideline 2023). With the established HCC surveillance and biannual ultrasound examination there is the possibility of early detection of iCCA. A recent metaanalysis shows, that around 1 in 4 patients with CCA has chronic liver diseases and 1 in 10 has liver cirrhosis underscoring the need for close surveillance strategies also for iCCA in the setting of prevention and early detection (Tham et al. 2024).

### Hepatolithiasis

Intrahepatic lithiasis is a major risk factor in the development of iCCA: 5–10% of patients with hepatolithiasis educe iCCA (OR 5–50) (Li et al. 2012). Additionaly the existence of choledochuscyst/Caroli-syndrome comes with a significant increase in iCCA incidence (relative risk 26.7) (Clements 2020). Regarding further predisposing cholestatic diseases we refer to the present EASL CPG for genetic cholestatic liver diseases (EASL Guideline cholestatic liver disease 2024).

## Diagnostic and staging

### Imaging

Suspicion of iCCA is often first raised by ultrasound. Further evaluation should be performed with MRI (or CT), with MRI being superior for assessing intrahepatic tumor spread (ESMO Guideline 2023, EASL-ILCA Guideline 2024, Deutsche S3-Leitlinie).

Unlike HCC, iCCA has less distinct features on contrast-enhanced imaging. Small iCCA lesions in cirrhosis can be misinterpreted as HCC and in the sclerotic subtype of HCC, typical imaging characteristics may be absent, raising the suspicion of iCCA (Deutsche S3-Leitlinie). In patients with apparently resectable iCCA, FDG-PET should routinely be performed to detect lymph node metastases (EASL-ILCA Guideline 2023).

### Tumor-biopsy

The gold standard of diagnosis is histology. Immunhistochemistry can support the diagnosis of CCA; however, no specific marker constellation is pathognomonic (Deutsche S3-Leitlinie). Positivity for K7, K19 and CA-19.9 suggests pancreato-biliary differentiation, and may help distinguish CCA from extrahepatic metastases. A key clinical challenge is differentiation from so-called adenocarcinoma of unknown primary (Adeno-CUP) (Paradis 2019). In cases where no extrahepatic primary tumor (e.g., pancreas, lung, breast) can be identified, an unrecognized iCCA may be the underlying cause. Comprehensive histological, immunohistochemical, and molecular pathological work-up is therefore essential (Deutsche S3-Leitlinie).

Tumor-biopsy is recommended to establish a definitive diagnosis. Identification of iCCA-subtypes (see above) relies on histological evaluation. Additionaly, the detection of actionable molecular targets is essential to guide potential second-line treatment options (Khan et al. 2012; Bridgewater et al. 2014). As chronic liver disease is a risk factor for iCCA, biopsy can also provide information on the non-tumorous liver parenchyma, which has therapeutic and prognostic implications (Deutsche S3-Leitlinie). In patients with potentially resectable disease and a pre-test high probability of iCCA, biopsy may be omitted; in such cases, the firm diagnosis and molecular characterization can be performed on the resected specimen.

### Molecular- pathological work-up

Current guidelines of the European Society for Medical Oncology (ESMO) recommend molecular characterisation of CCA by next generation sequencing (NGS) in all patients. The use of the ESCAT-classification (Scale for Clinical Actionability of Molecular Targets) is advised to guide clinical decision-making (Table 3).

**Table 3:** ESCAT (Scale for Clinical Actionability of Molecular Targets) – classification: Molecular characterization and classification of cholangiocarcinoma, adapted from ESMO Guideline 2023 and Mosele et al

**ESCAT I**	Alterations with already established therapy (z.B. IDH-1; FGFR-2-fusion)
ESCAT II	Alterations in clinical testing (z.B. BRAF V600E mutations)
ESCAT III	Alterations like HER2, that are already potential targets in other tumor entities

In up to 30–40% of patients with iCCA, potentially targetable molecular alterations can be identified. Given the limited efficacy of current second-line therapies, international guidelines recommend performing molecular diagnostic early – ideally at the time of initial diagnosis (Verdaguer et al. 2022, Benson et al. 2021).

## Therapy

**Fig. 2:**
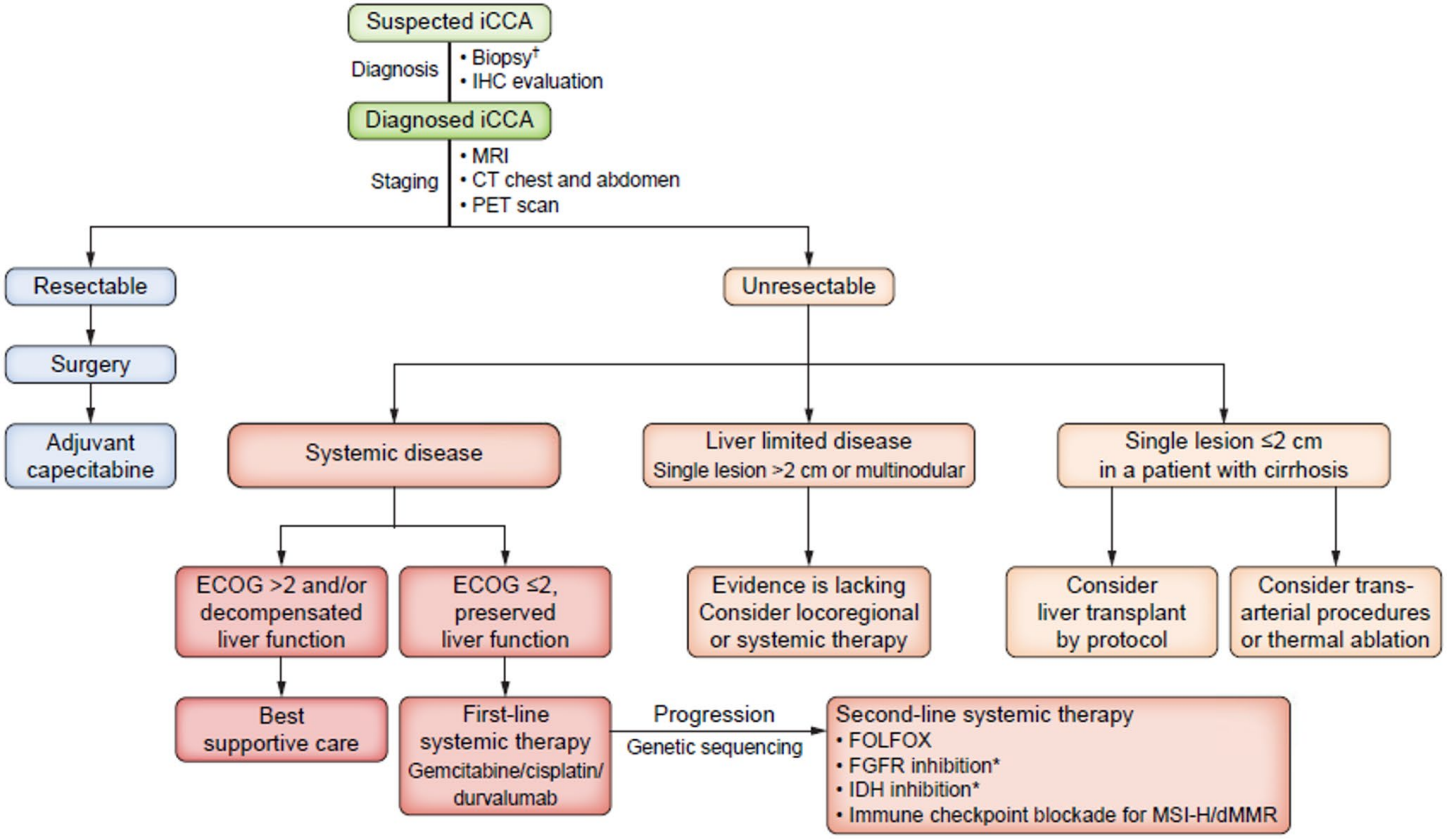
Algorithm of diagnostic and management of intrahepatic cholangiocarcinoma. Secondary resection after neoadjuvant therapy should be considered. FGFR, fibroblast growth factor receptor, FOLFOX, fluoruracil/oxaliplatin; ICC, intrahepatic cholangiocarcinoma; IDH Isocitratdehydrogenase; PET, Positron-Emission-Tomography (Figure taken from EASL-ILCA-Guideline 2023). 3 Reprinted with permission by Elsevier (Ref.Nr. 6101370428404)

The management of iCCA includes curative, adjuvant, neo-adjuvant and palliative strategies, selected according to tumor stage and anticipated treatment tolerance (Fig. 2). As iCCA is most often diagnosed at an advanced stage and given the complexity of the disease, patients should be referred to specialized oncology centers. All cases should be discussed in interdisciplinary tumor boards to ensure optimal use of available diagnostic and therapeutic options.

## Surgery

Surgery with regional lymphadenectomy is the only modality that can cure BTC. Resection with no tumor at the margin (R0) is the aim, as survival rates are higher than R1 or R2 status (ESMO Guideline 2023, EASL-ILCA Guideline 2023). Surgery involving hepatic resection should also consider the future liver remnant (FLR): patients without a fundamental disease of the liver parenchyma FLR should be 25–30%, whereas in patients with chronic liver disease FLR should be at least 40% to avoid postoperative liver failure (Mazzaferro 2020). An interventional preoperative tool to augment the expected FLR is the unilateral embolization of the portal vein or the transarterial radioembolziation (TARE) of the thereafter resected liver lobe. Postoperative mortality after major-resections can be reduced significantly (Glantzounis et al. 2017). After R0 resection 5-year survival rates range from 20 to 45%. Lymphnode involvement in PET-CT is no contraindication for resection, a lymphadenectomy of at least 3 lymph nodes is recommended (Sahara et al. 2019). Isolated intrahepatic recurrence of iCCA can be treated by resection, if a R0 situation is achievable (Deutsche S3-Leitlinie 2024).

Multiple studies emphasise the importance of pathology in assessing prognosis (ESMO Guideline 2023). Tumor number and size, surgical resection with microscopic tumor margin (R1 resection), nodal involvement and microvascular invasion are recognised negative prognostic factors in patients undergoing resection (Kim et al. 2015; Lamarca et al. 2021; Spolverato et al. 2015). Post hoc analysis of the Advanced Biliary tract Cancer (ABC)-01, -02 and -03 studies revealed an increase in median overall survival (OS) of ca. 4 months in patients with iCCA compared with non-iCCA BTCs, suggesting that iCCA has a more favourable natural history (Lamarca et al. 2020; ESMO Guideline 2023).

## Transplantation

Liver transplantation is not considered a standard treatment for iCCA and participation in clinical trials should be encouraged (ESMO Guideline 2023). In small iCCA confined only to the liver, which are not resectabel due to liver function impairment, liver transplantation can be considered. Available data show in patients with liver cirrhosis, tumor size < 3 cm, no lymphnode involvement and good grading 5-year survival of 73% (EASL-ILCA Guideline 2023). Owing to missing randomized trials regarding this research question, these patients should be treated in randomized prospective studies (Deutsche S3-Leitlinie 2024).

### Neoadjuvant and adjuvant therapy

Patients with primarily irresectable iCCA can benefit from neoadjuvant therapy if tumor response reaches resectability. Preliminary results of neoadjuvant chemotherapy versus direct resection in patients with CCA show promising data for neoadjuvant chemotherapy with improved OS and R0 resection rate (Goetze et al. ASCO annual meeting abstract 2025). Multimodal therapy approaches with combination of systemic therapy (congruent to systemic therapy in the first line setting) and locoregional therapies can be tried (ESMO Guideline 2023).

The high 3-year recurrence rate (up to 80%) after curative-intent resection for BTC has forced international study groups to investigate adjuvant therapy (ESMO Guideline 2023; Mavros et al. 2014; Tsilimigras et al. 2020).

To date three negative randomised controlled trials (RCTs) evaluated different adjuvant chemotherapy regimens compared with surgery alone. The Japanese BCAT study (gemcitabine), the French PRODIGE-12 study (gemcitabine and oxaliplatin) and the UK Bilcap study (capecitabine) (ESMO Guideline 2023, Primrose et al. 2019; Edeline et al. 2019; Ebata et al. 2018). The studies reported no significant improvement in OS in the intent-to-treat (ITT) population; however, in the predefined per protocol analysis of the BILCAP study, median OS was significantly improved with capecitabine compared with observation (53 months versus 36 months, respectively; adjusted hazard ratio ((HR) 0.75, 95% CI 0.58–0.97, p = 0.028). Long-term data of the BILCAP study have recently been published: the median OS was 49.6 months in the capecitabine-arm and 36,1 month in the observation arm (Bridgewater 2022). The ASCOT trial in Japan, which included a similar patient population to BILCAP, demonstrated that adjuvant therapy with four 6-weekly cycles of tegafuregimeracile oteracil (S1; an orally acting fluoropyrimidine) led to significantly longer survival than surgery alone ((HR) 0.694, 95% CI 0.514–0.935, p = 0.008) (Ikeda et al. 2022). All patients with resection in curative intend should be offered adjuvant therapy despite the before mentioned limitations.

### Adjuvant radiotherapy

The data supporting adjuvant radiotherapy (RT) are limited and not available for iCCA. A multicentre phase II study (SWOG S0809) included 79 patients with extrahepatic CCA or GBC (Ben-Josef et al. 2015). Patients had undergone radical resection and received gemcitabine and capecitabine followed by chemoradiotherapy with capecitabine. The primary objective of the study was met; as the level of evidence in iCCA is limited, RT is not routinely used in the adjuvant setting in iCCA treatment.

## Palliative systemic therapy and targeted therapies

### First-line therapy

Given that most patients are diagnosed at an advanced stage, the decision to initiate systemic treatment should be made a multidisciplinary tumorboard due to the complexicity of disease presentation. The therapeutic arsenal includes systemic chemotherapy, and immunotherapy, targeted agents, as well as locoregional modalities including radiation (ESMO Guideline 2023; EASL-ILCA Guideline 2023; Deutsche S3-Leitlinie 2024).

Two immune checkpoint inhibitor–based regimens have emerged as standard first-line options in advanced iCCA, both in combination with **gemcitabine and cisplatin** (GemCis): **durvalumab** (anti–PD-L1) and **pembrolizumab** (anti–PD-1). In the absence of head-to-head comparisons, both regimens are considered equally valid.

The TOPAZ-1 trial demonstrated that the addition of durvalumab to GemCis significantly improved overall survival (OS), progression-free survival (PFS), and objective response rate (ORR) compared to chemotherapy alone (Oh et al. 2022). Similarly, the KEYNOTE-966 trial showed a significant OS benefit for the combination of pembrolizumab with GemCis versus GemCis alone (Kelly 2023).

### Second-line therapy

The current standard of care in the second line setting is **FOLFOX** (folin-acid, 5-fluoruracil and oxaliplatin). The randomised controlled phase III trial ABC-06 demonstrated a statistically significant improvement in overall survival compared to best supportive care (BSC) (6.2 months vs. 5.3 months) (Lamarca 2021). This benefit was accompanied by a higher incidence of treatment-related adverse events, particularly neutropenia and fatigue.

### Molecular alterations

**Fig. 3:**
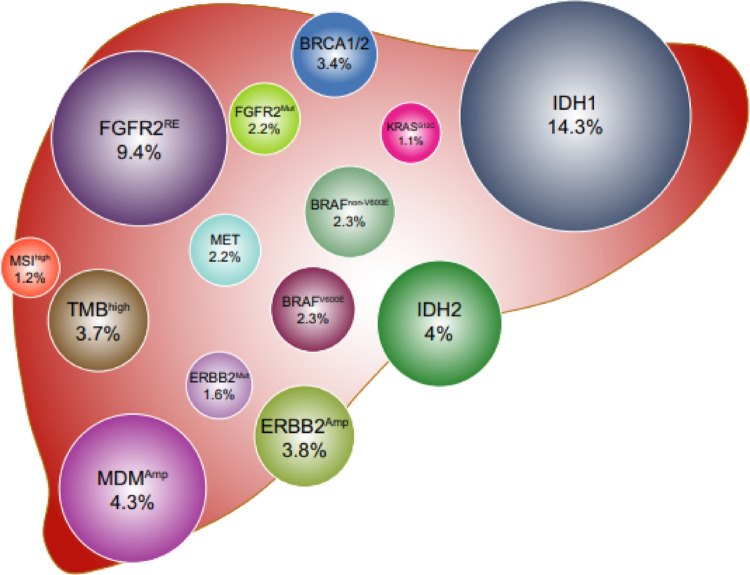
Occurence of possible molecular targets in intrahepatic cholangiocarcinoma (taken from Kendre et al.). Reprinted with permission by Elsevier (Ref.Nr. 6,101,370,255,004)

Advances in the molecular characterization of iCCA have identified a range of recurrent genetic alterations with potential diagnostic and therapeutic relevance. Frequently mutated or amplified genes include ARID1A, BAP1, EPHA2, FGFR2, IDH1, IDH2, KRAS, MCL1, PTEN, PTPN3, und TP53 (EASL-ILCA Guideline 2023). These alterations contribute to the dysregulation and hyperactivation of key oncogenic signaling pathways (Fig. 3).

Several of these alterations are clinically actionable: in particular, IDH1/2 mutations and FGFR2 fusions have led to the development of targeted therapies with a shift towards biomarker-driven treatment strategies in iCCA (Table 4).

**Table 4:** Frequency of actionable molecular alterations in iCCA and therapeutic (bold letters with EMA-approval, adapted from Deutsche S3-Leitlinie 2024).^4^

Molekular alteration	Frequency (%)	Therapeutic option(s)
RAS-mutation	10–20	–
TP53-mutation	20–30	–
FGFR2-translocation	15–30	**Pemigatinib, futibatinib,** infigratinib
IDH1/2	10–20	**Ivosidenib**
BRAF V600E	3–6	Dabrafenib and trametinib
MSI-H (MLH1,MSH2,MSH6,PMS2)	1–2	**Pembrolizumab**
NTRK1-3	< 1	Larotrectinib, entrectinib
HER 2	Up to 20	Trastuzumab + pertuzumab

### FGFR-2 fusion and rearrangement

Genomic alterations involving fibroblast growthfactor receptor 2 (*FGFR2)* are found in up to 15% of patients with iCCA. Following progression on first line therapy, selective FGFR2 inhibitors such as **pemigatinib, infigratinib and futibatinib** have demonstrated promising efficacy in single-arm phase II trials. Progression-free survival (PFS) ranged from 6.9 to 9 months and OS from 12.2 to 21.7 months (Abou-Alfa 2020; Goyal 2023; Javle 2021). Objective response rates (ORR) were 35.5% for pemigatinib, 23.1% for infigratinib, and 41.7% for futibatinib. Pemigatinib was the first agent in this class to receive regulatory approval from both FDA and EMA. To date, no randomized trials have compared FGFR2 inhibitors directly with FOLFOX or other second-line regimens. Targeted therapies generally show a more favorable tolerability profile compared to conventional cytotoxic chemotherapy.

HER2/neu (human epidermal growth factor receptor 2) alterations represent a predicitive biomarker and a promising therapeutic target in up to 10% of cholangiocarcinomas and up to 20% of gallbladder carcinomas (ESMO Guideline 2023). HER-2-targeted therapies, established in other tumour entities (e.g. breast cancer or gastric cancer) are now being investigated in biliary tract cancers, including iCCA.

In a phase II trial, zanidatamab—a bispecific anti-HER2 antibody—achieved an objective response rate (ORR) of 41.3% (Pant 2023). Comparable activity was observed with the combination of tucatinib (a selective HER2 tyrosine kinase inhibitor) and trastuzumab, yielding an ORR of 46.7% (Nakamura 2023). In the MyPathway basket trial, HER2 blockade with pertuzumab and trastuzumab resulted in an ORR of 23% and a median overall survival of 10.9 months (Javle 2021).

Although HER2-directed therapy has not yet received regulatory approval for iCCA, the current ESMO guideline recommends the combination of trastuzumab and pertuzumab as a second line option in HER2-positive disease (ESMO Guideline 2023).

### IDH1-gene mutation

Mutations in the IDH1-gene are found in approximately 10–28% of patients with iCCA (EASL-ILCA Guideline 2023). In the randomized phase 3 trial (CLarIDHy), the selective IDH1 inhibitor **ivosidenib** significantly improved progression-free survival (PFS) compared to placebo (2.7 vs. 1.4 months; *p* < 0.001) and showed a numerical overall survival (OS) benefit (10.3 vs. 7.5 months; *p* < 0.01) (Abou-Alfa). Ivosidenib was overall well tolerated, and current evidence supports its use in the second line setting for patients with *IDH1*-mutated tumors.

### Further treatment options second-line

For patients with microsatellite instability-high (MSI-H) iCCA who have not previously received PD-(L)1 blockade, the PD-1 inhibitor pembrolizumab is EMA-approved as a second-line option (EASL-ILCA Guideline 2023).

In approximately 5% of patients with biliary tract cancers, *BRAF V600E* mutations are detectable**.** The combination of the BRAF inhibitor **dabrafenib** and the mitogen-activated protein kinase (MEK) inhibitor **trametinib** demonstrated an objective response rate (ORR) of 51% and a median overall survival (OS) of 14 months in the ROAR basket trial (Subbiah 2020). This regimen is FDA-approved but not yet approved by the EMA (ESMO Guideline 2023).

Fusions involving *NTRK* genes are rare (< 0.1%) in biliary tract cancers. Targeted therapy with the NTRK inhibitors larotrectinib or entrectinib may be considered (ESMO Guideline 2023).

## Locoregional therapy/local ablation

The management of patients with non-metastatic unresectable iCCA should be guided by a multidisiplinary team (MDT).

### Ablational therapies

Stereotactic body radio therapy (SBRT) achieves local tumor control but is associated with a modest OS rate; a 1-year OS rate of 58.3% has been reported (ESMO Guideline 2023). SBRT can be considered in patients with liver-confined tumor burden and no surgical (ESMO Guideline 2023).

Thermal ablation techniques, including radiofrequency, microwave and cryoablation, have shown efficacy in small tumors. A systematic review reported a pooled complete ablation rate of 93% and a median OS of 30.2 months (Edeline et al. 2021). Ablation is most effective in lesions < 3 cm that are not suitable for resection (ESMO Guideline 2023). In selected cases, ablation may also be combined with transarterial chemoembolization (TACE) for tumors up to 5 cm with curative intent (Deutsche S3-Leitlinie 2024). Emerging data suggest that in carefully selected patients, e.g., tumors within Milan criteria and with adequate peritumoral ablation margins, thermal ablation may offer outcomes comparable to resection (Pang 2024).

### Intraarterial therapies

TACE and TARE are established locoregional treatment options for iCCA (EASL-ILCA Guideline 2023). A systematical review reported a median OS of 12.4 months following TARE, despite extrahepatic disease in 33% of patients (Yang et al. 2015). Downstaging with subsequent R0 resection has been described in selected cases (Al-Adra et al. 2015). Recent data suggest improved OS when TARE is combined with systemic therapy (Edeline et al. 2024).

Other intra-arterial approaches include hepatic arterial infusion (HAI) of chemotherapy (mainly melphalan) and chemosaturation, a technique that delivers higher intrahepatic melphalan doses with extracorporeal filtration. The potential benefit of chemosaturation is currently being investigated in clinical trials (Vogel et al. 2017; Marquardt et al. 2019).

**Author contribution**

Aaron Schindler: Conceptualization, investigation, writing original draft, review and editing. Timm Denecke: Data curation, resources, review and editing. Daniel Seehofer: investigation, review and editing. Florian van Bömmel: Conceptualization, writing original draft, review and editing. Thomas Berg: Conceptualization, investigation, review and editing. All authors read and approved the final manuscript.

## Data Availability

No datasets were generated or analysed during the current study.
